# Thermally Conductive Shape Memory Polymer Composites Filled with Boron Nitride for Heat Management in Electrical Insulation

**DOI:** 10.3390/polym13132191

**Published:** 2021-06-30

**Authors:** Andrzej Rybak, Lukasz Malinowski, Agnieszka Adamus-Wlodarczyk, Piotr Ulanski

**Affiliations:** 1 ABB Corporate Technology Center, Starowislna 13A, 31-038 Krakow, Poland; lukasz.malinowski@pl.abb.com; 2 Faculty of Chemistry, Institute of Applied Radiation Chemistry, Lodz University of Technology, Wroblewskiego 15, 93-590 Lodz, Poland; agnieszka.adamus@p.lodz.pl (A.A.-W.); piotr.ulanski@p.lodz.pl (P.U.)

**Keywords:** shape memory, polymer composites, thermally shrinkable materials, thermal conductivity, electrical insulation

## Abstract

The evaluation of a possible application of functional shrinkable materials in thermally conductive electrical insulation elements was investigated. The effectiveness of an electron beam and gamma radiation on the crosslinking of a selected high density polyethylene grade was analyzed, both qualitatively and quantitatively. The crosslinked polymer composites filled with ceramic particles were successfully fabricated and tested. On the basis of the performed investigation, it was concluded that the selected filler, namely a boron nitride powder, is suitable for the preparation of the crosslinked polymer composites with enhanced thermal conductivity. The shape memory effect was fully observed in the crosslinked samples with a recovery factor reaching nearly 99%. There was no significant influence of the crosslinking, stretching, and recovery of the polymer composite during shape memory phenomenon on the value of thermal conductivity. The proposed boron nitride filled polyethylene composite subjected to crosslinking is a promising candidate for fabrication of thermally shrinkable material with enhanced heat dissipation functionality for application as electrically insulating components.

## 1. Introduction

The shape memory effect (SME) is a phenomenon in which a specially prepared functional material recovers its original dimension and shape when heated above a specific transformation temperature. Shape memory polymers (SMP) possess two forms: a temporary shape which is maintained before the actual use of the material, and the second memorized form which is named as a permanent shape. The methods commonly used for fabrication of permanent forms are chemical and physical crosslinking processes applied to thermoplastic or thermosetting polymers [1]. The SMP material can be transformed from a permanent shape into a temporary form by means of additional processing through heating, then subsequent deformation, and cooling at the last stage. The SME polymer preserves the temporary form until the appearance of the adequately defined external stimulus which triggers the transformation of the current shape into the permanent form [2].

SMP materials are commonly used for application as heat shrinkable electrical insulation components, such as heat shrink sleeves for cable joints and terminations, shrinkable molded shapes, or simple shrinkable tubes for cable repairs [3,4]. Due to the flow of the electrical current through electrification components, as in cables and busbars, additional heat is generated, which can lead to malfunctioning or even the damaging of the electrical devices. Therefore, suitable thermal management in order to dissipate the excess of generated heat is a crucial issue for appropriate functionality of electrical apparatuses [5,6,7,8,9,10,11,12,13].

Polyethylene is a commonly used material as electrical insulation due to its adequate dielectric, thermal, and mechanical properties [14,15,16,17]. One can find in the literature a large number of studies showing the heat shrinkable behavior and the physicomechanical and structural properties of electron beam [18,19,20,21] or gamma radiation [22,23,24] crosslinked high-density polyethylene. Pure polymers exhibit a very low thermal conductivity, and the heat dissipation from the internal parts of electrical components to the outside is highly hindered. One of the approaches to enhance the thermal conductivity of polymer materials is the application of high thermally conductive fillers [5,6,7,8,9,10,11,12,13]. There are studies regarding the fabrication of heat shrinkable polymer composites with different fillers incorporated, such as carbon nanotubes (CNT) [25], nanoclay [26], glass fiber [27], and silicon carbide [28,29]. However, there is not enough research on the fabrication of SMPs with enhanced thermal conductivity that, at the same time, maintain electrical insulation.

Therefore, the aim of the presented work was to evaluate an SMP which can be used for thermal management in electrical devices. The first part of the research work was done in order to confirm the possibility of the crosslinking of a specific type of polyethylene using ionizing radiation, both in the form of electron beam and gamma rays. The crosslinking process efficiency was assessed by both qualitative and quantitative approaches (assessment of gel fraction as a function of absorbed dose). The ability of a polymer to efficiently form crosslinks upon irradiation and to form a substantial crystalline fraction is a prerequisite for manufacturing thermo-shrinkable products using a radiation technique.

The aim of the second part of the work was to crosslink the boron nitride powder-filled polyethylene samples in the shape of a rectangular bar by ionizing radiation, using doses which are expected to turn the substrates into materials possessing detectable and usable thermo-shrinkable properties. The performed investigations have proven that the obtained SMP material exhibits both high shape recovery and enhanced thermal conductivity and is suitable for heat management in electrical insulation.

## 2. Materials and Methods

### 2.1. Materials Used

#### 2.1.1. Polymer Matrix

Polyethylene type Hostalen GC 7260 (supplied by LyondellBasell, Ludwigshafen, Germany) was selected for the investigation. Hostalen GC 7260 is a high-density polyethylene (HDPE) resin used in general injection molding applications for electrification devices. Hostalen GC 7260 demonstrates good flowability and high rigidity. The typical properties are:

- Density: 0.960 g/cm^3^;
- Tensile Modulus: 1450 MPa;
- Tensile Stress: 30 MPa;
- Vicat Softening Temperature: 74 °C;
- Polymer has a form of white pellets with diameter of ca. 3 mm.

#### 2.1.2. Filler

For the purpose of the presented investigation, the CoolFX CFX-1026 boron nitride (BN) powder, supplied by Momentive, was used as a filler. The BN is a well-known additive used in industrial applications [10]. The main benefit from using BN in thermal management applications is that thermal conductivity can be increased while maintaining electrical insulation which is crucial for application of SMPs in electrification devices [13].

### 2.2. Crosslinking of Pellets

The samples containing ca. 5 g each of HDPE were placed in small, flat envelopes of polyethylene foil, degassed, and vacuum sealed. Polymer pellets were crosslinked by means of two types of irradiation methods: electron beam and gamma rays.

#### 2.2.1. Electron Beam (EB) Irradiation

The samples were placed separately in central positions of flat cardboard boxes of 40 × 40 × 3 cm and located on an automated vertical conveyor system in front of the electron beam exit. The samples were irradiated by a horizontally swept beam of 6 MeV electrons from a linear electron accelerator ELU-6 (Elektronika) [30,31,32]. Alanine and calorimetric dosimeters were used to set the desired absorbed doses of 25, 50, 75, and 100 kGy. While no precise dose mapping was made for these irradiations, data from other analogous procedures on the same equipment and using similar sample geometry show that the average dose should not vary by more than 5% from the target one, while the spatial inhomogeneity of the dose is typically below 15%.

#### 2.2.2. Gamma Ray Irradiation

The samples, in their individual vacuum-sealed envelopes, were irradiated in the radiation chamber by gamma rays of an average energy of 1.25 MeV emitted by cobalt-60 isotope from a panorama-type OB-Servo D irradiator (Izotop) [32]. The dose rate was ca. 3 kGy/h as determined by alanine dosimetry.

After irradiation, the samples were kept in the vacuum-sealed bags until needed for testing. All tests were performed within 2 weeks after irradiation.

### 2.3. Characterization of HDPE Pellets

#### 2.3.1. Thermal Properties and Crystallinity

Thermal properties and crystallinity of the nonirradiated material were assessed by differential scanning calorimetry (DSC). Approximately 5 mg of the sample was placed in a standard aluminum pan and analyzed on a Q200 DSC calorimeter (TA Instruments, New Castle, DE, USA) under nitrogen atmosphere using a cycle of heating (from 40 °C to 150 °C), cooling (from 150 °C to 80 °C), and heating (from 80 °C to 150 °C), at a constant temperature gradient of 10 deg/min.

Degree of crystallinity (χ_c_) was calculated by relating the experimentally determined enthalpy of melting (ΔH_m_) and cold crystallization (ΔH_cc_) of the analyzed sample to the known enthalpy of melting of a fully crystalline polyethylene (291 J/g), according to the following formula [33,34]:

χ_c_ = ΔH/291 J·g^−1^. (1)

#### 2.3.2. Qualitative Crosslinking Tests

Qualitative tests on crosslinking were performed by placing ca. 0.5 g of the sample in a glass test tube filled with 20 mL of xylene, heating it on an oil bath to boiling temperature, and keeping it at or slightly below boiling temperature for 1 h. The observations were made while the sample was still hot. Hazy appearance or presence of visible, swollen, but insoluble gel particles was taken as an evidence of gel fraction formation.

#### 2.3.3. Quantitative Crosslinking Tests

The exact known amount (ca. 0.5 g) of the sample was placed in a glass test tube, 20 mL of xylene was added, and the tube content was kept for 1 h at or slightly below the boiling temperature of xylene. A stainless-steel mesh filter of submillimeter mesh size was preheated to the same temperature. The hot content of the test tube was poured onto the mesh, leading to the separation of the swollen HDPE pellets. This was immediately followed by washing the mesh content several times with a total of ca. 50 mL of boiling xylene to wash out the unbound polymer fraction. The as-washed swollen pellets were subsequently dried to a constant weight to remove the solvent. The ratio of their dry weight to the initial weight of the sample was taken as the gel fraction.

### 2.4. Manufacturing of BN Filled HDPE Composites

#### 2.4.1. Samples Preparation

The same HDPE Hostalen GC 7260 which was used for preliminary crosslinking tests was also selected for fabrication of unfilled and BN-filled composite samples. In order to increase thermal conductivity of polymer samples, the selected fractions of BN powder were added.

**Figure 1:**
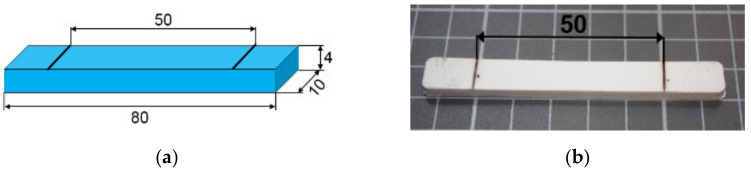
(**a**) Scheme of investigated rectangular samples; and (**b**) the image of fabricated bars with a marked central area width of 50 mm which was subjected to stretching.

First HDPE pellets filled with BN were prepared by means of extrusion process with single screw extruder (ZAMAK EHP-25E, Skawina, Poland), and then the rectangular bars were prepared by use of laboratory injection molding machine (ZAMAK WT 12, Skawina, Poland). Figure 1 shows dimensions of the produced samples. 

The following types of the rectangular samples were prepared:

- Pure HDPE;
- HDPE + 35 wt.% BN;
- HDPE + 55 wt.% BN.

#### 2.4.2. Crosslinking of Rectangular Composite Samples

In order to limit access of oxygen to the samples during and after irradiation, and thus to prevent excessive oxidation, each sample was vacuum sealed in an individual pouch of PE foil. Each pouch was marked to identify the kind of sample and the dose to be delivered.

Gamma irradiation was selected as a tool to induce crosslinking. While the alternative method, i.e., EB irradiation, has the advantage of high-speed processing, gamma irradiation offers almost unlimited possibilities of treating samples of various, even complex, geometry and size. Taking into account future applications of the tested materials in the form of elements of various shapes and sizes, gamma irradiation seems to be more versatile and universal for this purpose than electron beam treatment.

The samples, in their individual vacuum-sealed pouches, were gamma irradiated as described in Section 2.2.2 at a dose rate of 3.8 kGy/h.

Four samples of each kind were irradiated with a dose of 100 kGy, and the remaining four with a dose of 150 kGy (see Table 1 with list of samples). The choice of doses was based on the results achieved in Section 3.2, indicating that the gelation dose for this kind of HDPE was in the order of 50 kGy, and also on the analysis of the literature [18,19,20,21,22,23,24,25,26,27,28,29], where doses ranging from 100 up to several hundred kGy were typically used. The chosen doses were in the lower part of this range, because it is expected and reported in some of the previous works that too high of a degree of crosslinking may make the product difficult to be expanded at high temperatures (which is the next step of formation of a thermo-shrinkable part), and the product may exhibit only a low degree of shrinkage when heated during the final application.

### 2.5. Shape Memory Tests

**Figure 2:**
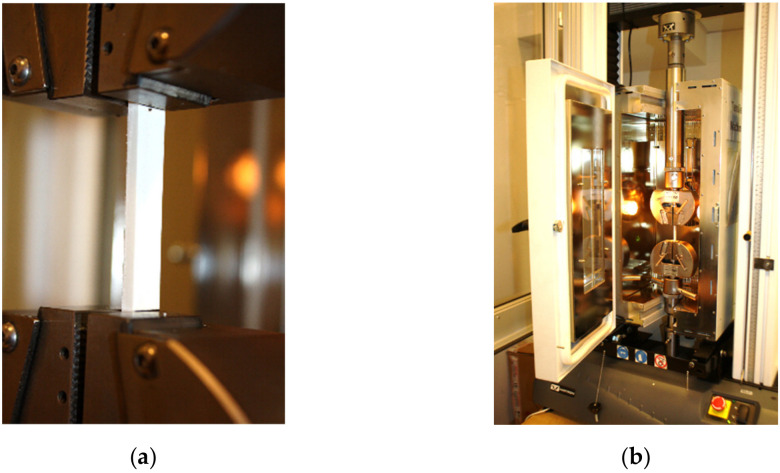
(**a**) Sample fixed between holders of tensile machine and (**b**) sample inserted into the oven. The length of the visible area of sample was equal to 50 mm.

The cross-linked rectangular samples (as shown in Figure 1) were subjected to the following procedure in order to investigate the shape memory effect:

1. At the beginning, all the samples were subsequently fixed between holders of the tensile machine Instron 3367 and closed in oven as shown in Figure 2.
2. Then, samples were heated at 120 °C for 15 min and stretched by 10%, in such a way that the area of 50 mm indicated in Figure 1 and visible in Figure 2a was elongated to the value of 55 mm.
3. In order to freeze the sample in elongated state and create the temporary forms of the investigated SMP, the samples were still kept stretched and cooled to reach room temperature, and then removed from holders: hereinafter referred to as a 1st stage.
4. The change of stretched distance was measured after 1st stage.
5. As the next step, the free-standing samples were heated again to temperature of 125 °C for 15 min and cooled to room temperature: hereinafter referred to as a 2nd stage.
6. Finally, the recovery of the stretched length after 2nd stage was measured.

A detailed description with a schematic representation of the formation and action of a thermo-shrinkable material based on polyethylene can be found in the literature [1,2]. For each type of composite, four samples were investigated, and the average values were calculated.

### 2.6. Thermal Conductivity Measurements

The thermal conductivity of composites was evaluated with Hot Disk method on Hot Disk TPS 500 instrument (Hot Disk AB, Gothenburg, SWEDEN), where output power was 0.3 W and time of measurement was 20 and 2.5 s for unfilled and filled samples, respectively. The number of four samples was investigated for each sample type. Considering the shape of the sample, the thermal conductivity measurement was performed in the perpendicular direction to the injection molding direction. The measuring sensor was placed between two composite samples, in contact with the sample side with the largest area.

## 3. Results and Discussion

### 3.1. Thermal Properties and Crystallinity of the Non-irradiated HDPE

**Figure 3:**
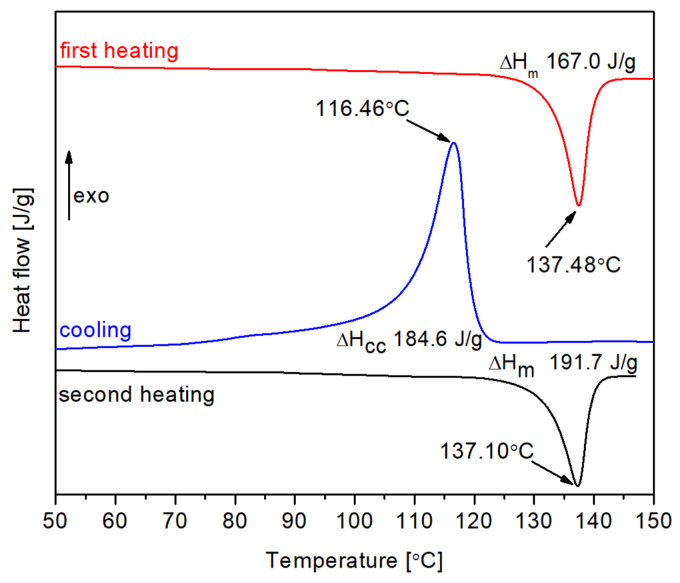
DSC thermograms of nonirradiated HDPE. The sample was cycled through heating, cooling, and heating at a temperature gradient of 10 deg/min under nitrogen atmosphere.

Thermograms resulting from DSC analysis of a nonirradiated HDPE pellet are presented in Figure 3. The samples were subjected to a heating-cooling-heating cycle. The characteristic melting peak is observed for ca. 137 °C at heating and ca. 116 °C at cooling, clearly indicating the presence of a crystalline phase. The integrating of these peaks allows the degree of crystallinity to be calculated. The obtained values were 57.4%, 63.4%, and 65.9% for the first heating, cooling, and second heating, respectively. While the first heating value depends on the thermal history of samples, the cooling and second heating values indicate that it is easy to reach crystalline fraction of ca. 65%.

The ability of HDPE to form a substantial number of crystallites is important for the planned application as a thermo-shrinkable material. It should be stressed that there may be some changes in crystallite formation ability caused by irradiation, although it is not expected to be a strong effect if moderate doses are applied. Moreover, the degree of crystallinity can be different from the values determined here if crystallization is induced by cooling in a stressed condition, as expected in the procedure of forming thermo-shrinkable applications.

### 3.2. Qualitative Crosslinking Verification

A visual inspection of the irradiated samples has been performed. There were no noticeable changes in physical form of the granulate, except for a slight yellowish coloration progressing with irradiation dose, occurring both in samples irradiated by electron beam and gamma rays. This is a typical effect, which may be caused by:

- formation of a low amount of unsaturated bonds;
- some oxidation;
- presence of residual (trapped) free radicals in the crystalline fraction.

Due to the generally low amplitude of those effects, they are not expected to interfere to a large extent with the thermo-shrinkable properties of the crosslinked HDPE.

Linear polyethylene is soluble in boiling xylene, while the crosslinked fraction (gel fraction) is not. The visual testing of the presence of a crosslinked fraction (insoluble in boiling xylene) yielded results presented in Table 2.

While there is no evidence for any insoluble fraction in the nonirradiated sample and in the samples irradiated with a dose of 25 kGy, traces of insoluble material can be seen in the 50 kGy sample (slight turbidity), while a clearly distinguishable gel fraction (in the form of swollen, but not dissolved, “cores” of the HDPE pellets) was detected in samples irradiated with doses of 75 and 100 kGy.

### 3.3. Quantitative Estimation of Crosslinking

**Figure 4:**
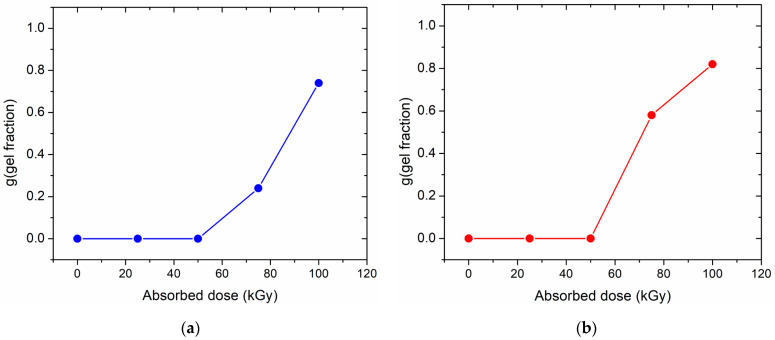
(**a**) Estimated gel fractions of HDPE as a function of absorbed dose for samples irradiated with electron beam and (**b**) gamma rays.

Results of the quantitative estimation of the gel fraction in the irradiated samples are presented in Figure 4.

While it should be stressed that the presented values are approximate due to the relative simplicity of the chosen test method, a clear tendency is observed in both cases, i.e., electron beam and gamma ray irradiation. Irradiation predominantly causes crosslinking of the studied HDPE samples. At a dose in the order of 50 kGy, there is an onset of gel fraction, i.e., a macroscopically observable crosslinked, insoluble polymer. The gel fraction increases with absorbed dose. For the highest dose applied in our tests, i.e., 100 kGy, the gel fraction reaches approximately 80%, which is a considerably high level. Within the accuracy margin of these tests, no substantial difference can be seen between the effects of electron beam and gamma ray irradiation.

The observed behavior is typical for irradiation of “crosslinking-type” polymers, including polyethylene. Quantitative results may vary with the physicochemical parameters of the irradiated HDPE samples and with irradiation conditions; however, when these parameters are constant, one should expect relatively good reproducibility.

It may be of some interest to discuss, in the present context, the possible influence of irradiation on PE chemical and physical structure, in particular its crystallinity, as well as the influence of the BN on the crosslinking of PE. 

Ionizing radiation causes the ionization and excitation of macromolecules. Apart from the very early stages of the radiation-induced events, the main chemical step is based on the formation and subsequent reactions of polymer-derived radicals. There are three main processes induced by ionizing radiation in solid polymers: crosslinking, chain scission (degradation), and oxidation [35,36,37]. Since both gamma and EB irradiations in this study were performed with no oxygen access and the minor amount of oxygen present in the volume of the samples must have been used up in the first few kGy of irradiation, in practical terms, we can neglect the latter process in our discussion. When irradiated in the absence of oxygen, polyethylene is known to undergo mainly crosslinking. The ratio between the yields of crosslinking and scission in polyethylene is typically over 3 [36], and while the exact number may depend on several factors, a radiation synthesis of crosslinked PE is a standard and well-established technology. Irradiated samples are not expected to differ significantly in their chemical structure from the parent polymer. Some unsaturation can be formed as a result of a side process of radical disproportionation. Moreover, some radicals formed in the crystalline phase may become trapped there [38]. Both these effects, together with a very minor number of oxidized groups formed by the radical reactions with traces of oxygen initially present in the sample volume, may contribute to the slight off-while coloration (see Section 3.4). The yield of these products under conditions used in this work is expected to be low, and their presence is of marginal importance with respect to the product properties. 

The physical structure does change due to the 3D network formation by crosslinking. At the gelation dose (ca. 50 kGy under the conditions of our study), on average, one crosslink will be formed for each polymer chain originally present in the system [36,39]. Of course, there is a distribution of this number (magnified by the distribution of chain lengths); some chains would have more than one crosslink, while others would have none. At 100 kGy, the average number of crosslinks per chain would be ca. 2, the distribution resulting from ca. 20% of chains still being unbound. The final structure is further complicated by the high crystallinity of the starting material (ca. 57%). While the crystallites themselves act as physical crosslinks, they contain less chemical (radiation-induced) crosslinks than the amorphous phase, due to the higher mobility of chain segments and thus higher tendency of radicals to recombine in the latter [40]. 

While radiation-induced crosslinking can influence the ability of the polymer to recrystallize after the melting of the crystalline phase due to the imposed geometrical constraints [41], moderate doses of radiation absorbed at room temperature (i.e., below the melting point) are not expected to have pronounced effects on the degree of crystallinity. A reduction in crystallinity is observed at doses much higher than used in this work [42]. What is interesting is that the presence of boron nitride as a filler has been observed to mitigate the radiation-induced reduction in crystallinity [43].

The presence of inorganic fillers in principle may influence the outcome of polymer irradiation in at least three ways. If the filler contains atoms of a high atomic number Z or is of very high density, it is expected to attenuate ionizing radiation, which may cause uneven dose distribution in irradiated objects. With boron nitride (low Z, moderate density of 2.1 g/cm^3^) and gamma irradiation or EB, this effect is expected to be small (in contrast to neutrons, which are efficiently stopped by BN [44,45]), particularly if the highly penetrating gamma rays are used to irradiate PE objects of a moderate thickness (many cm). Secondly, the presence of nano- or microparticles can make the polymer matrix formed by crosslinking somewhat denser, with the particles serving as additional crosslinking points. Finally, there may be some influence of radiation chemistry of the filler itself (which may be manifested by the effect mentioned in Section 3.4 below), but for inorganic particles, this is expected to be of moderate importance.

### 3.4. Effect of Irradiation on Appearance of Samples

A visual inspection of the irradiated samples (shown in Figure 1) has been performed. There were no noticeable changes in physical form of the rectangular bars, except for slight yellowish/brownish coloration, similar to what was observed in the preliminary tests on pellets. This is a typical effect accompanying irradiation of polyethylene, which was already discussed in Section 3.2.

An interesting effect was that the presence of the additive seemed to limit the extent of observable color change. While studying this effect was beyond the scope of this work, one may expect that the additive might:

- change the crystalline structure and degree of crystallinity of HDPE during the bar manufacturing step (which may result, e.g., in a lower tendency to trap free radicals in the crystalline region);
- act to some extent as a radioprotective agent;
- interfere with irradiated HDPE by partial transfer and/or scavenging of radicals.

One cannot also exclude that the difference is mainly a physical effect of changing the transparency of the specimens and thus changing the apparent color intensity after irradiation.

### 3.5. Shape Memory Phenomenon

The shape memory effect was studied according to the procedure described in Section 2.5. For all the samples, the initial central area width and stretching distance were the same, 50 and 55 mm, respectively. The investigated area lengths, after both heating and cooling stages, were recorded. The obtained measurement results are presented in Table 3.

It worth pointing out that after the 1st stage, in which the samples were kept stretched and cooled to room temperature, the samples removed from the holders already recovered ca. 1.9 to 2.4 mm of the stretched 5.0 mm. The reason for the observed effect, namely a not fully frozen temporary shape, was the relaxation of the induced mechanical stresses during the stretching of the sample.

One can see that after the 2nd stage in which the free-standing samples were heated to a temperature of 125 °C for 15 min and again cooled to room temperature, the investigated area of the sample has shortened again by ca. 2.4 mm. Finally, at the end of the heating and cooling procedures, a final length close to the initial one was recovered (cf. Table 3), and a clear indication of the shape memory effect was fully observed.

From the comparison of the shape recovery of the samples crosslinked with different absorbed doses, namely for HDPE 100 vs. HDPE 150, as well as HDPE + 35BN 100 vs. HDPE + 35BN 150, one can see that the higher the dose, the better the efficiency of the shape recovery. This can be explained by the fact that stronger irradiation caused a higher degree of polymer chain crosslinking, therefore resulting in the shape memory phenomenon being more noticeable. A similar range of obtained high shape recovery efficiencies were also reported in the literature for polyethylene composites filled with different fillers: CNT [25], nanoclay [26], glass fiber [27], and silicon carbide [28,29].

It is worth pointing out that it was not possible to perform stretching for the samples with 55 wt.% BN content. Due to high filler loading, cracking was initiated, and it was not possible to obtain good quality samples for shape memory effect investigation.

### 3.6. Thermal Conductivity Measurements

**Figure 5:**
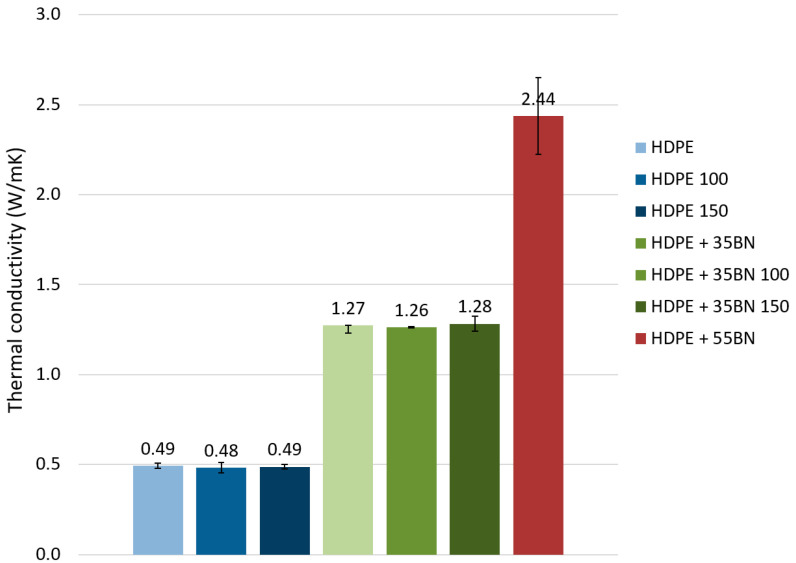
Thermal conductivity values measured perpendicularly to injection molding direction for samples with different content of the BN filler: 0, 35, and 55 wt.%. Results are shown for reference pristine samples and samples after testing shape memory effect. Bars with standard deviation are indicated for each type of specimen. See Table 1 for sample definitions.

The unfilled and filled crosslinked samples, stretched and after recovery to their initial size, and also the reference untreated samples (named HDPE, HDPE + 35BN, and HDPE + 55BN) were investigated in order to evaluate the influence of the shape memory effect on thermal conductivity. The obtained results are collected in Figure 5.

One can clearly see that thermal conductivity for the samples with the same BN content, measured before crosslinking and after the shape memory effect, is very similar. Therefore, it can be concluded that there is no significant influence of the crosslinking, stretching, and recovery to initial size during the shape memory phenomenon on the value of thermal conductivity. The obtained values of thermal conductivity for pure and filled HDPE samples are in the range observed in the literature for BN-filled polymer composites [13].

As was mentioned, the measurements of thermal conductivity were performed in a perpendicular direction to the injection molding direction. It is well known that during the injection molding process, the fillers are forced to align along the injection direction, and as a result, the formation of the conductive paths is also preferred in this direction. Therefore, the measured thermal conductivity perpendicular to the injection molding direction indicates the lower limit, and one can expect that the thermal conductivity evaluated in the parallel direction, namely along the injection molding, should give higher thermal conductivity [46]. It is worth pointing out that the effect of polymer chain stretching at the filler interface on the molecular structure and anisotropy of the thermal conductivity was revealed in the molecular simulations [47]. It was shown that the stretched polymer layers between the confining surfaces of the fillers possess an anisotropic heat conduction, namely the heat transfer in the direction parallel to the surfaces is much higher than that in the perpendicular direction [48].

Due to the earlier described problem of cracking during the stretching of the sample with 55 wt.% BN content, it was not possible to measure the thermal conductivity of samples after the shape memory effect. However, one can expect that there is a potential for the optimization of samples in order to evaluate samples with maximum filler content that still show a good level of shape memory effect. This will allow the most effective cooling parts suitable for application as electrical insulation to be created. Such a statement can be derived on the basis of the high thermal conductivity observed for the sample with 55 wt.% BN loading before being subjected to stretching, as shown in Figure 5.

## 4. Conclusions

The obtained crosslinking tests have indicated that the selected type of polyethylene can be crosslinked using ionizing radiation, both in the form of electron beam and gamma rays, in the absence of air. The onset of macroscopic gel fraction formation occurs at ca. 50 kGy, while at 100 kGy, substantial amounts of gel (in the order of 80%) are present. The tested polymer is capable of forming a crystalline fraction, with a degree of crystallinity of ca. 65% achieved by cooling under static, stress-free conditions.

The shape memory effect was clearly observed in the crosslinked polyethylene samples filled with boron nitride, resulting in a very high percentage of shape recovery to the initial state (close to 99% in the case of HDPE filled with 35 wt.% of BN and gamma irradiated with 150 kGy dose). The slight influence of the filler addition and the crosslinking conditions on the shape recovery efficiency was observed.

The selected filler, namely boron nitride powder, is suitable for the preparation of polymer composites with enhanced thermal conductivity. The addition of 35 wt.% of BN leads to a 161% enhancement of the thermal conductivity for the investigated shape memory composite. No significant influence of the crosslinking, stretching, and recovery of size during the shape memory phenomenon on the value of thermal conductivity was observed. The proposed composite can be used for the fabrication of thermally shrinkable parts with heat dissipation functionality for electrification devices.

## Figures and Tables

**Table 1:** List of rectangular specimens prepared with different BN content and irradiation dose.

Sample Name	BN Content (wt.%)	Gamma Irradiation Dose (kGy)
HDPE	0	-
HDPE 100	0	100
HDPE 150	0	150
HDPE + 35BN	35	-
HDPE + 35BN 100	35	100
HDPE + 35BN 150	35	150
HDPE + 55BN	55	-
HDPE + 55BN 100	55	100
HDPE + 55BN 150	55	150

**Table 2:** Solubility of HDPE samples in boiling xylene. Presence of insoluble material (gel fraction) is marked by “+”, whereas “−” indicates absence of crosslinked residues.

Dose (kGy)	0	25	50	75	100
Electron beam	-	-	+/− ^1^	+	+
Gamma rays	-	-	+/−	+	+

^1^ “+/−” traces of insoluble material were slightly visible.

**Table 3:** Changes of the investigated area length (initial, stretched, and after 1st and 2nd stages) for different types of samples. Percentage of recovery to initial shape length is indicated in the last row.

Area Length ^1^ (mm)	HDPE 100	HDPE 150	HDPE + 35BN 100	HDPE + 35BN 150
Initial	50.00	50.00	50.00	50.00
Stretched	55.00	55.00	55.00	55.00
After 1st stage	52.61	52.55	53.19	53.07
After 2nd stage	50.18	50.11	50.81	50.68
Shape recovery ^2^ (%)	99.64	99.78	98.41	98.66

^1^ Length of central area of rectangular sample indicated in Figure 1. ^2^ Effectiveness of the shape memory effect was calculated as percentage of the ratio between initial length and final length after 2nd stage.

## Data Availability

The data presented in this study are available on request from the corresponding author.
